# A novel 14-gene signature for overall survival in lung adenocarcinoma based on the Bayesian hierarchical Cox proportional hazards model

**DOI:** 10.1038/s41598-021-03645-6

**Published:** 2022-01-07

**Authors:** Na Sun, Jiadong Chu, Wei Hu, Xuanli Chen, Nengjun Yi, Yueping Shen

**Affiliations:** 1 Department of Epidemiology and Biostatistics, School of Public Health, Medical College of Soochow University, Suzhou, 215123 China; 2 Department of Biostatistics, School of Public Health, University of Alabama at Birmingham, Birmingham, AL 35294 USA

**Keywords:** Prognostic markers, Risk factors

## Abstract

There have been few investigations of cancer prognosis models based on Bayesian hierarchical models. In this study, we used a novel Bayesian method to screen mRNAs and estimate the effects of mRNAs on the prognosis of patients with lung adenocarcinoma. Based on the identified mRNAs, we can build a prognostic model combining mRNAs and clinical features, allowing us to explore new molecules with the potential to predict the prognosis of lung adenocarcinoma. The mRNA data (*n* = 594) and clinical data (*n* = 470) for lung adenocarcinoma were obtained from the TCGA database. Gene set enrichment analysis (GSEA), univariate Cox proportional hazards regression, and the Bayesian hierarchical Cox proportional hazards model were used to explore the mRNAs related to the prognosis of lung adenocarcinoma. Multivariate Cox proportional hazard regression was used to identify independent markers. The prediction performance of the prognostic model was evaluated not only by the internal cross-validation but also by the external validation based on the GEO dataset (*n* = 437). With the Bayesian hierarchical Cox proportional hazards model, a 14-gene signature that included CPS1, CTPS2, DARS2, IGFBP3, MCM5, MCM7, NME4, NT5E, PLK1, POLR3G, PTTG1, SERPINB5, TXNRD1, and TYMS was established to predict overall survival in lung adenocarcinoma. Multivariate analysis demonstrated that the 14-gene signature (HR 3.960, 95% CI 2.710–5.786), T classification (T_1_, reference; T_3_, HR 1.925, 95% CI 1.104–3.355) and N classification (N_0_, reference; N_1_, HR 2.212, 95% CI 1.520–3.220; N_2_, HR 2.260, 95% CI 1.499–3.409) were independent predictors. The *C*-index of the model was 0.733 and 0.735, respectively, after performing cross-validation and external validation, a nomogram was provided for better prediction in clinical application. Bayesian hierarchical Cox proportional hazards models can be used to integrate high-dimensional omics information into a prediction model for lung adenocarcinoma to improve the prognostic prediction and discover potential targets. This approach may be a powerful predictive tool for clinicians treating malignant tumours.

## Introduction

Lung cancer is one of the most common cancers in the world and is the leading cause of cancer-related deaths^1^. With the aging of the global population, lung cancer has a critical impact on health worldwide. Furthermore, lung adenocarcinoma is an important lung cancer subtype that has attracted increasing attention from researchers^2,3^. Due to the 5-year survival rate of lung adenocarcinoma being comparatively low, thus, improving its clinical prognosis is one of the main goals of clinical workers and medical researchers. Most of the previous prognostic models of lung adenocarcinoma focused on the clinical factors, such as treatment, tumour node metastasis (TNM) stage, and tumour grade^4,5^. These models may not be able to accurately predict the survival of patients with lung adenocarcinoma.

With the development of molecular technologies, we have the opportunity to integrate high-dimensional omics information into a prediction model of lung adenocarcinoma to improve its prognostic prediction ability, discover potential therapeutic targets and guide clinical treatment. This has become a new strategy to predict the prognosis of patients with lung adenocarcinoma^6–8^. In previous studies, the most common analysis strategy focused on selecting the most significant differential expression genes first, performing least absolute shrinkage and selection operator (LASSO) regression to calculate a risk score from high-dimensional omics data, and using Cox regression analysis to combine the risk score with clinical factors to establish an effective prognosis model^9,10^. To a certain extent, these model has a higher *C*-index than the prognosis models that only contain clinical factors^11^.

However, the gradual development of the Bayesian method provides new ideas for research in this field and is recognized by an increasing number of scholars. Bayesian statistics is a kind of statistical inference based on population, sample, and prior information. In this context, Yi et al. combined Bayesian statistics with the classical LASSO Cox regression model and constructed a new prediction model, the Bayesian hierarchical Cox proportional hazards model, which obtained a higher *C*-index and had better stability^12^. More importantly, the expectation–maximization (EM) cyclic coordinate descent algorithm is used to fit the model, which increases the speed of the analysis. Up to now, the Bayesian hierarchical Cox proportional hazards model has not been applied to the prognosis and prediction of high-dimensional omics in lung adenocarcinoma.

In this study, the Bayesian hierarchical Cox proportional hazards model was applied to reduce the dimensionality of the transcriptomics data and explore the mRNAs related to the prognosis of lung adenocarcinoma. An independent prognostic factor was constructed involving a 14-gene prognostic signature based on a data set from The Cancer Genome Atlas (TCGA). Multivariate Cox proportional hazard regression was then used to build the final prediction model, combined with the risk score and clinical characteristics, and a prognostic nomogram was constructed for clinical application. In addition, the stability of the model was verified using the Gene Expression Omnibus (GEO) data set.

## Material and methods

### Study cohort

#### TCGA data sets

The mRNA data and clinical data for lung adenocarcinoma samples from the TCGA-LUAD data set were obtained from the TCGA database^13^. The mRNA data sets consisted of normal samples (*n* = 59) and lung adenocarcinoma samples (*n* = 535). Additionally, the following clinical information was obtained: age, gender, race, T classification, N classification, M classification, stage, treatment, smoking history, survival status, and overall survival (OS). After excluding the samples from patients with missing values, more than 10 years of follow-up, and an OS time of fewer than 15 days, samples from a total of 470 patients were selected for the study cohort.

#### GEO data sets

The GEO database provides the largest available set of microarray data with clinical annotation for lung adenocarcinoma. The gene expression profiling data sets for the GSE68465 cohort were downloaded from the GEO database for validation studies^14^. The genetic and clinical data for 443 patients with lung adenocarcinoma were obtained and taken into account the aforementioned inclusion and exclusion criteria, 437 patients were selected for the validation cohort.

TCGA and GEO belong to public databases. The patients involved in the database have obtained ethical approval. Users can download relevant data for free for research and publish relevant articles. Our study is based on open-source data, so there are no ethical issues and other conflicts of interest.

### Gene set enrichment analysis (GSEA)

GSEA^15^ mainly uses genomic and gene sequencing to detect biological differences in microarray data sets^16^. In this study, critical pathways and leading-edge mRNAs in lung adenocarcinoma versus normal control samples were identified by GSEA, using the Molecular Signatures Database (MSigDB) c2 (c2.cp.kegg.v7.2.symbols.gmt)^17^. The false discovery rate (FDR) < 0.25, nominal *P* value < 0.05, and |Normalized Enrichment Score (NES)| > 1 were regarded as the criteria for the identification of significant pathways^18^.

### Statistical analysis

#### Univariate Cox proportional hazards regression and Bayesian hierarchical Cox proportional hazards model

The univariate Cox proportional hazards regression was adopted for the initial dimension reduction of high-dimensional data. To explore the gene signatures potentially affecting the survival of lung adenocarcinoma patients, R version 4.0.2 software was used to analyze the data, and *P* < 0.05 was considered a statistically significant difference. The Bayesian hierarchical Cox proportional hazards model was used to establish the optimal multivariate model, and dimension reduction was realized by the bmlasso function through the R “BhGLM” package^19^. Moreover, the EM cyclic coordinate descent algorithm and spike-and-slab mixture double-exponential prior [formula (1)] were selected to fit the model^12^.

$${\beta }_{j}|{\gamma }_{j},{s}_{0},{s}_{1}\sim DE({\beta }_{j}|0,{s}_{j})=\frac{1}{2{s}_{j}}\mathit{exp}(-\frac{\left|{\beta }_{j}\right|}{{s}_{j}})$$

1 $${s}_{j}=(1-{\gamma }_{j}){s}_{0}+{\gamma }_{j}{s}_{1}$$

The spike scale value *s*_*0*_ and the slab scale value *s*_*1*_ cause strong or weak shrinkage of *β*_*j*_, respectively (0 < *s*_*0*_ < *s*_*1*_). Moreover, an initial value is required for the spike scale and the slab scale. Additionally, a previous study demonstrates that the spike scale value *s*_*0*_ has a strong influence on the model effectiveness, while the slab scale has little effect on the model effectiveness^20^. Therefore, in this study, we set the initial values as follows: *s*_*0*_ = *c* (*s*_*λ*_ − 0.05, *s*_*λ*_ − 0.04, *s*_*λ*_ − 0.03, *s*_*λ*_ − 0.02, *s*_*λ*_ − 0.01, *s*_*λ*_, *s*_*λ*_ + 0.01, *s*_*λ*_ + 0.02, *s*_*λ*_ + 0.03, *s*_*λ*_ + 0.04, *s*_*λ*_ + 0.05), *s*_*1*_ = 0.5, where *s*_*λ*_ is the optimal penalty of the LASSO Cox model. The concordance index (*C*-index) and the validation deviance were used to select the optimal model through tenfold with 10 repeats cross-validation^21^.

After building the optimal Bayesian hierarchical Cox proportional risk model, the genes with nonzero coefficients were selected to calculate the risk score [formula (2)].

2 $$risk\, scores= {\sum }_{j=1}^{n}coefj*Xj$$

where $coefj$ is the coefficient, $Xj$ is the standardized gene expression in the optimal model. After calculating the risk score for each patient, the median risk score was regarded as the cut-off value that stratified lung adenocarcinoma patients into low-risk and high-risk groups to compare the survival. The area under the curve (AUC) of each data set was calculated for detailed evaluations.

#### Multivariate Cox proportional hazards regression

**Figure 1:**
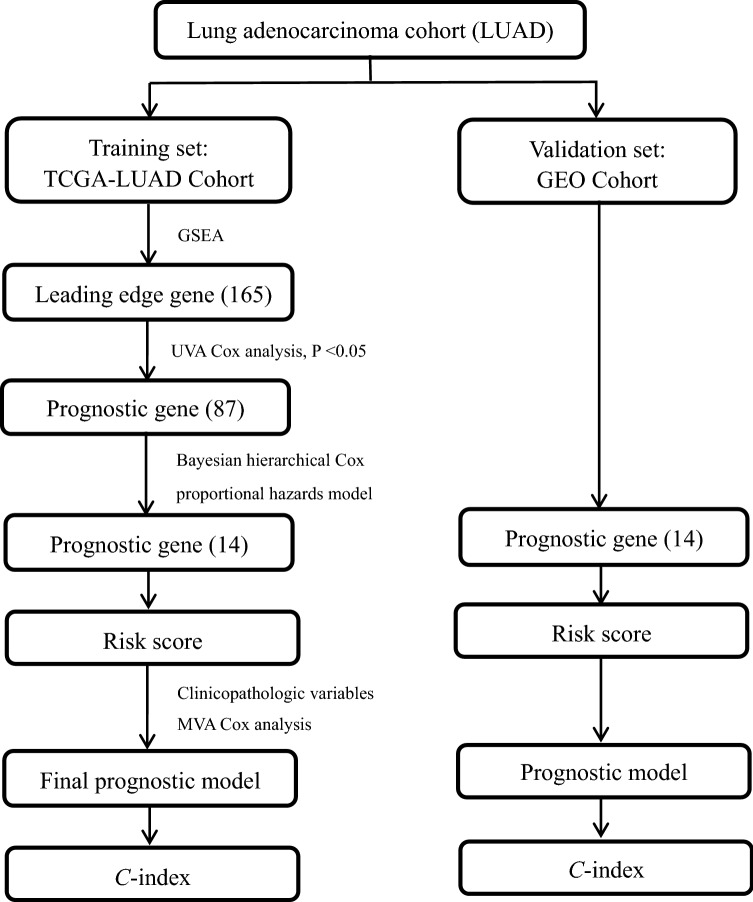
The workflow of this study.

Finally, we combined the risk score with clinical characteristics to construct the prognostic model. The results were sequentially displayed by a forest plot using the R package “forestplot”. In addition, the nomogram provided information on the relationship between the total points, risk score, and clinical characteristics to predict the 3-year, 5-year, 10-year overall survival rates for new patients. To ensure the stability of the results, the *C*-index obtained from 1000 bootstrap samples was used to measure the validity of the nomogram. Furthermore, we calculated the total point of each patient using the nomogram and divided the patients into two groups according to the median total point to compare the survival. Finally, calibration curves of the 3-year, 5-year, 10-year survival rates were drawn to verify the consistency of the overall survival rate data between the predicted values obtained using the nomogram and the actual values. The workflow of this study is shown in Fig. 1. I confirm that all methods were performed in accordance with the relevant guidelines and regulations.

## Results

The clinical characteristics of the TCGA-LUAD cohort and the GEO cohort are shown in Table 1. The results showed that the distribution of clinical characteristics in the two cohorts was comparable.

**Table 1:** Clinical characteristics of the lung adenocarcinoma cohort in the study.

Factor	TCGA	GEO
No. of patients	470	437
Age, years, mean (SD)	65.2 (10.01)	64.4 (10.10)
**Gender, no. (%)**		
Female	251 (53.40)	218 (49.89)
Male	219 (46.60)	219 (50.11)
**Race, no. (%)**		
White	371 (78.94)	289 (66.13)
Other	57 (12.13)	19 (4.35)
Unknown	42 (8.94)	129 (29.52)
**T classification, no. (%)**		
T_1_	160 (34.04)	149 (34.10)
T_2_	251 (53.40)	249 (56.98)
T_3_	42 (8.94)	28 (6.41)
T_4_	17 (3.62)	11 (2.52)
**N classification, no. (%)**		
N_0_	312 (66.38)	297 (67.96)
N_1_	91 (19.36)	87 (19.91)
N_2_	67 (14.26)	53 (12.13)
**M classification, no. (%)**		
M_0_	314 (66.81)	437 (100.00)
M_1_	21 (4.47)	0 (0.00)
MX	135 (28.72)	0 (0.00)
**Stage, no. (%)**		
I	253 (53.83)	–
II	114 (24.26)	–
III	76 (16.17)	–
IV	21 (4.47)	–
Unknown	6 (1.28)	–
Neither	0 (0.00)	316 (72.31)
**Treatment, no. (%)**		
Chemotherapy	242 (51.49)	43 (9.84)
Radiotherapy	228 (48.51)	20 (4.58)
Chemotherapy and Radiotherapy	0 (0.00)	44 (10.07)
Unknown	0 (0.00)	14 (3.20)
**Smoking history, no. (%)**		
No	63 (13.40)	48 (10.98)
Yes	389 (82.77)	296 (67.73)
Unknown	18 (3.83)	93 (21.28)

### Gene set enrichment analysis

**Figure 2:**
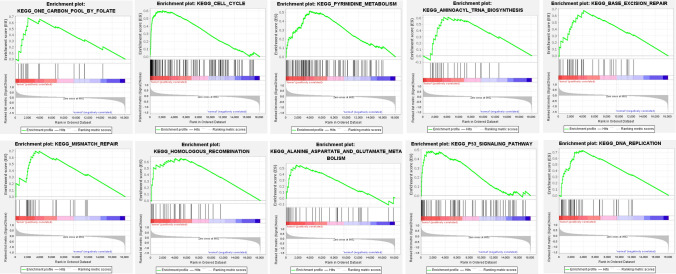
GSEA results from the c2 reference gene sets of the tumour group.

GSEA revealed that 10 pathways were involved in the tumour group. After removing the repeated genes in the pathways, 165 genes were identified for subsequent analysis. The details are shown in Table S1 and Fig. 2. In addition, the expression of these 165 mRNAs was visualised by a heatmap (Fig. S1).

### Prognosis-related mRNAs

**Figure 3:**
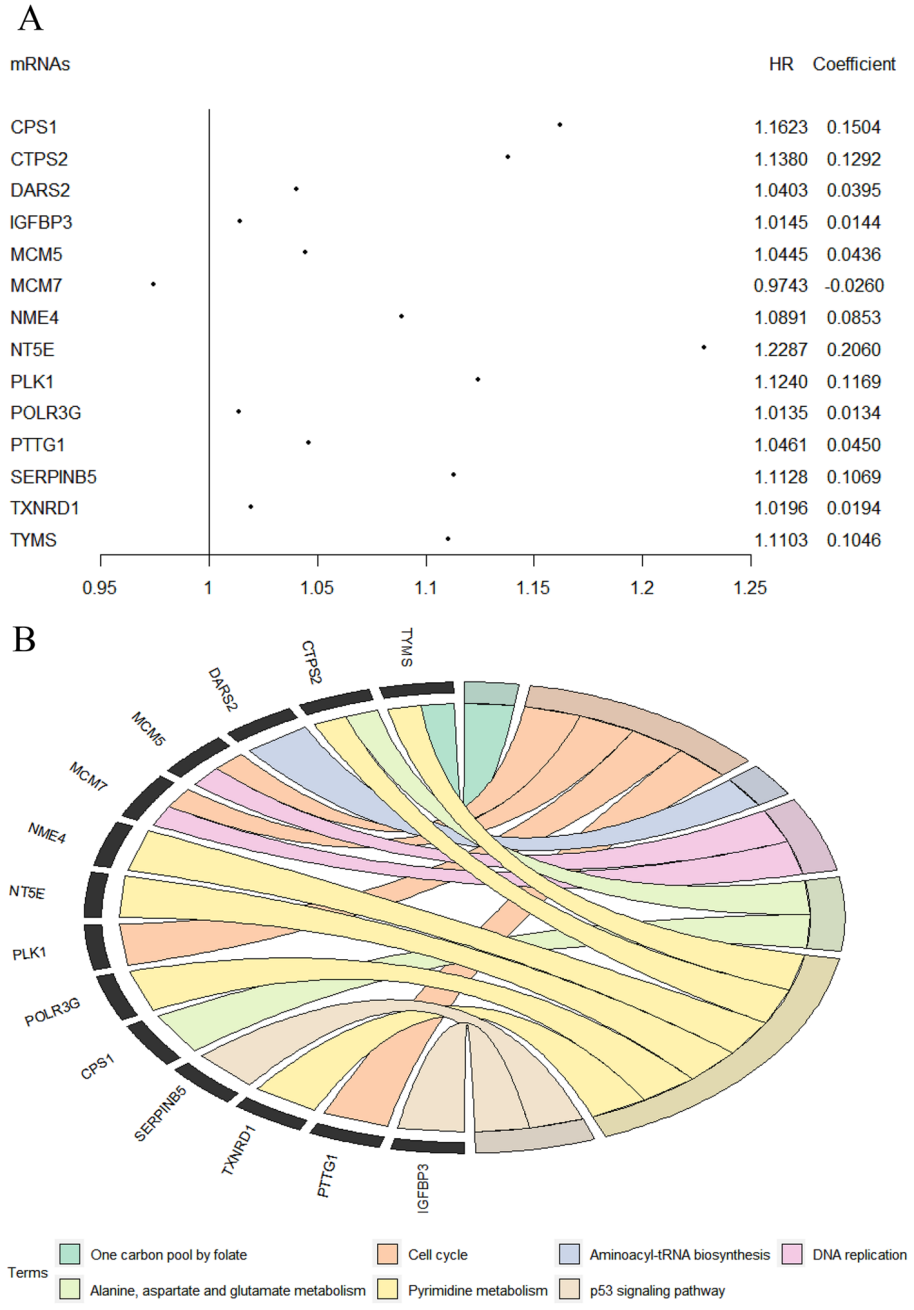
14-gene prognostic signature. (**A**) Estimate of HR for 14 genes using the Bayesian hierarchical Cox proportional hazards model with a spike-and-slab prior. (**B**) The chord diagram of prognosis-related mRNAs. Genes are represented on the left, and pathways are represented on the right. Different pathways are differentiated by different colours.

Univariate Cox proportional hazards regression analysis showed that 87 genes were related to the prognosis of lung adenocarcinoma. The LASSO Cox model was used to show that the optimal penalty *s*_*λ*_ = 0.0843. According to the mean of the *C*-index, we found that the prediction model was optimal when *s*_*0*_ = 0.0743 and *s*_*1*_ = 0.5 (Table 2). The *C*-index of the Bayesian hierarchical Cox proportional hazards model was 0.651, slightly higher than the *C*-index of the LASSO Cox regression, which was 0.649. In this model, we found that the following 14 genes were significantly related to patient survival: CPS1, CTPS2, DARS2, IGFBP3, MCM5, MCM7, NME4, NT5E, PLK1, POLR3G, PTTG1, SERPINB5, TXNRD1 and TYMS (Fig. 3A). The distribution of these 14 genes in each pathway was visually displayed by a chord diagram (Fig. 3B).

**Table 2:** The measurements of the optimal models for the TCGA lung adenocarcinoma (LUAD) dataset mRNAs by tenfold with 10 repeats cross-validation.

Method	*C*-index		Deviance	
	Mean	SD	Mean	SD
LASSO Cox	0.649	0.007	1779.056	4.590
*s*_*λ*_ − 0.05, 0.5	0.626	0.013	1787.719	6.311
*s*_*λ*_ − 0.04, 0.5	0.637	0.006	1786.711	3.539
*s*_*λ*_ − 0.03, 0.5	0.645	0.006	1781.919	2.940
*s*_*λ*_ − 0.02, 0.5	0.649	0.006	1779.648	3.111
s_λ_ − 0.01, 0.5	0.651	0.006	1779.030	3.328
*s*_*λ*_, 0.5	0.650	0.006	1779.095	3.984
*s*_*λ*_ + 0.01, 0.5	0.649	0.007	1779.092	4.659
*s*_*λ*_ + 0.02, 0.5	0.648	0.007	1779.666	5.335
*s*_*λ*_ + 0.03, 0.5	0.646	0.007	1780.872	5.847
*s*_*λ*_ + 0.04, 0.5	0.645	0.007	1782.788	6.259
*s*_*λ*_ + 0.05, 0.5	0.643	0.007	1785.146	6.803

Significant values are in bold.

*s*_*0*_ = *c* (*s*_*λ*_ − 0.05, *s*_*λ*_ − 0.04, *s*_*λ*_ − 0.03, *s*_*λ*_ − 0.02, *s*_*λ*_ − 0.01, *s*_*λ*_, *s*_*λ*_ + 0.01, *s*_*λ*_ + 0.02, *s*_*λ*_ + 0.03, *s*_*λ*_ + 0.04, *s*_*λ*_ + 0.05), *s*_*λ*_ = 0.0843.

**Figure 4:**
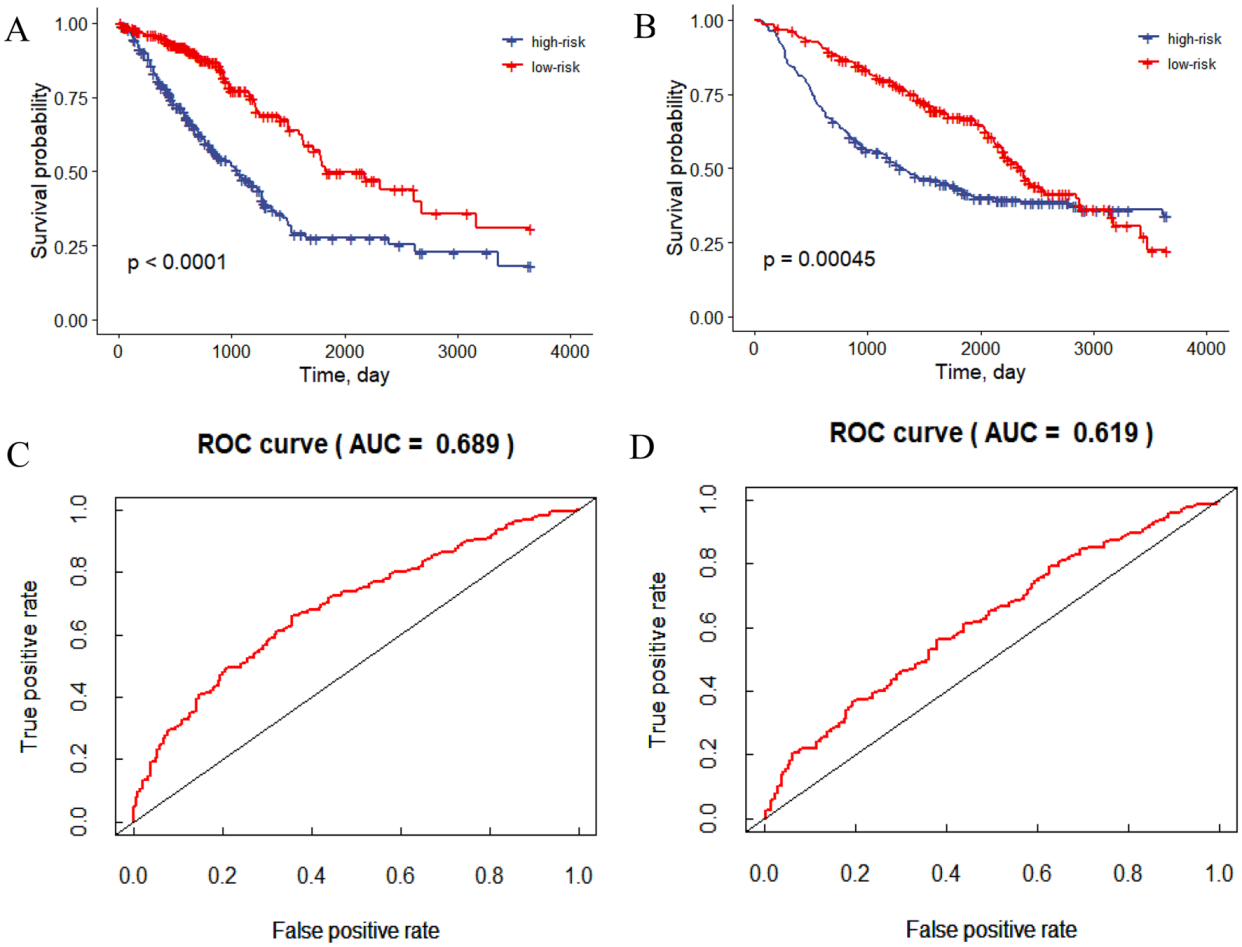
(**A**) Kaplan–Meier curve of TCGA-LUAD survival data for high-risk and low-risk groups with *P* < 0.001. (**B**) Kaplan–Meier curve of GEO survival data for high-risk and low-risk groups with *P* < 0.001. (**C**) The ROC curve of the risk score for predicting survival in the TCGA-LUAD Cohort. (**D**) The ROC curve of the risk score for predicting survival in the GEO Cohort.

**Figure 5:**
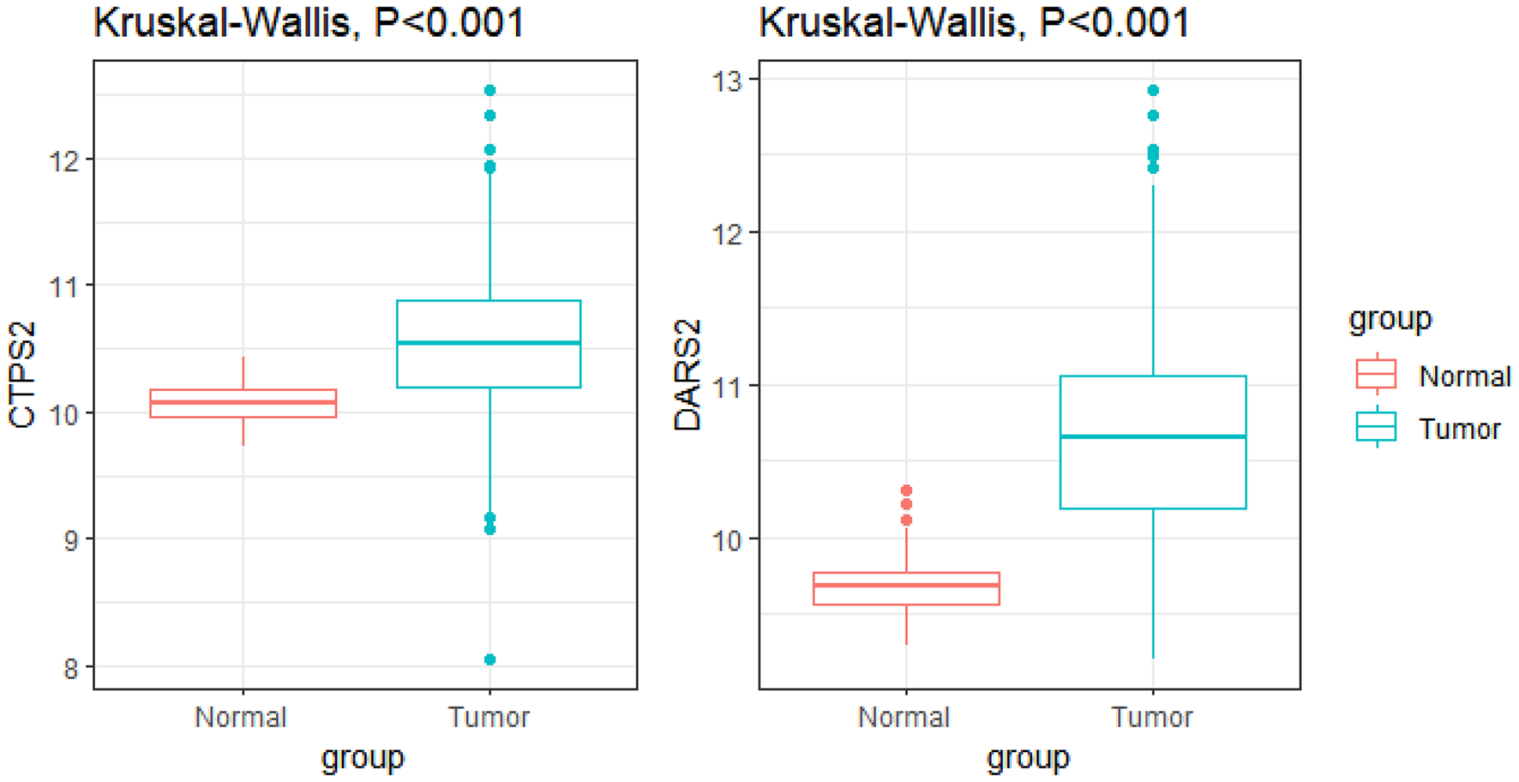
The CTPS2 and DARS2 expression in lung adenocarcinoma and normal groups in TCGA data. (**A**) The mRNA level of CTPS2 was dramatically increased in lung adenocarcinoma samples compared with normal lung samples. (**B**) The mRNA level of DARS2 was significantly higher in lung adenocarcinoma samples compared with normal lung samples.

We calculated the risk score for each patient and used the median risk score (median = − 0.068) to divide patients with lung adenocarcinoma into low-risk and high-risk groups. The Kaplan–Meier survival curve with log-rank test showed that patients with high-risk scores had shorter OS time than those with low-risk scores (*P* < 0.001, Fig. 4A), and the AUC of the risk score was 0.689 (Fig. 4C); similarly, the external validation set results were shown in Fig. 4B and D. Then, we analyzed the gene expression in lung adenocarcinoma and normal groups (CTPS2 and DARS2), which had not been fully explored. The results showed that the mRNA expression of CTPS2 was dramatically increased in lung adenocarcinoma samples compared with normal lung samples (*P* < 0.001, Fig. 5A). The mRNA level of DARS2 was significantly elevated in lung adenocarcinoma samples compared with normal lung samples (*P* < 0.001, Fig. 5B).

### Prognostic model

**Figure 6:**
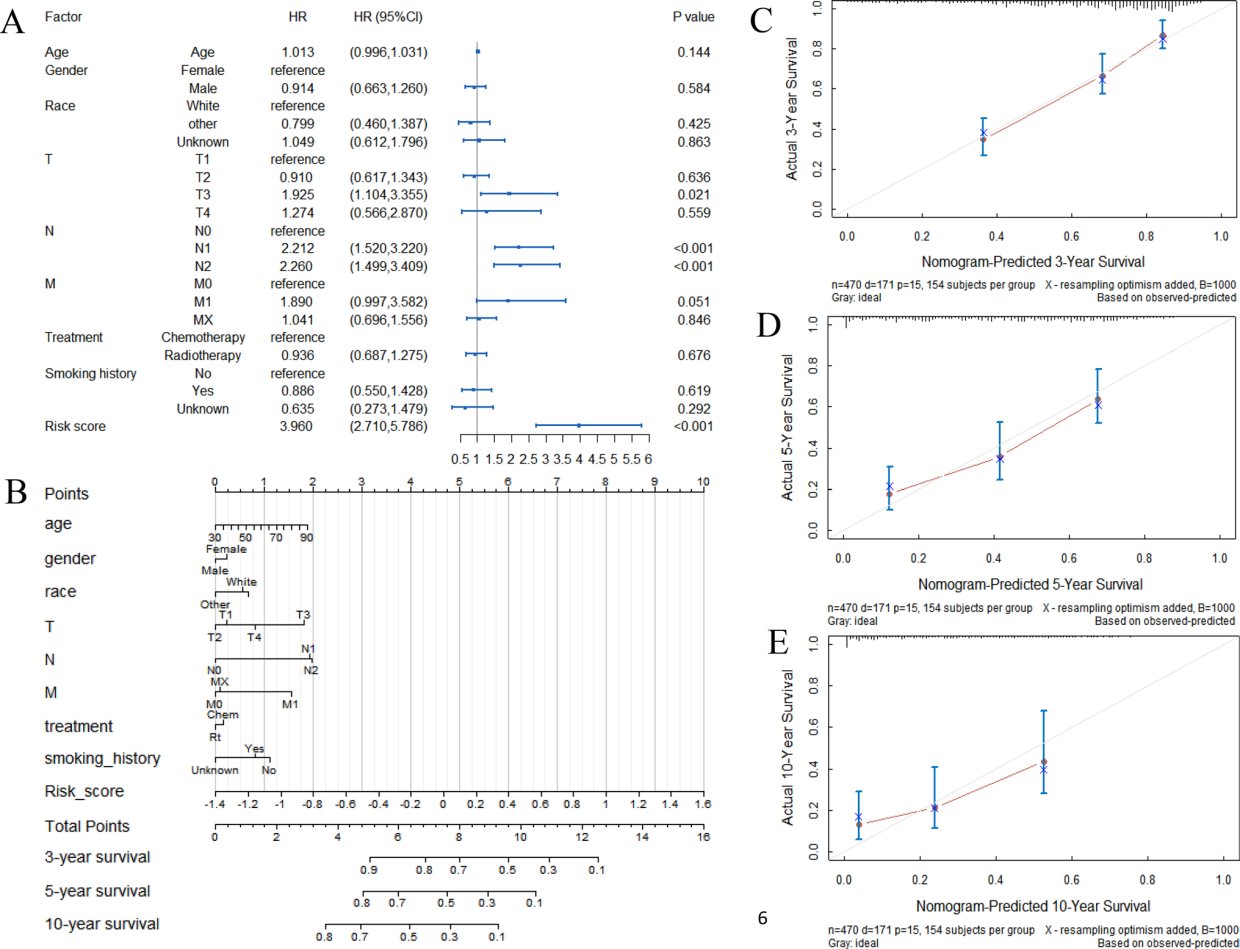
The final prognostic model. (**A**) Forest diagram of the risk score and clinical variables. (**B**) The nomogram for predicting the survival probability of lung adenocarcinoma patients at 3, 5 and 10 years. (**C**–**E**) Calibration curve of the nomogram for predicting 3-year, 5-year, and 10-year overall survival probability.

Multivariate Cox proportional hazards regression showed that the risk score (HR 3.960, 95% CI 2.710–5.786), T classification (T_1_, reference; T_3_, HR 1.925, 95% CI 1.104–3.355) and N classification (N_0_, reference; N_1_, HR 2.212, 95% CI 1.520–3.220; N_2_, HR 2.260, 95% CI 1.499–3.409) were independent predictors of lung adenocarcinoma patient survival (Fig. 6A). The *C*-indexes of the internal and external validation were 0.733 and 0.735, respectively. In addition, integrating the 14-gene signature and clinical factors, we generated a nomogram to predict the 3-year, 5-year and 10-year survival rates (Fig. 6B). Each factor was scored according to the proportion of its contribution to the survival rate. The Kaplan–Meier survival curve with log-rank test demonstrated that patients with high total points had shorter OS times than those with low total points (*P* < 0.001, Fig. S2). Calibration curves showed that there was consistency between the predicted and actual values (Fig. 6C–E), especially for the 3-year survival rate.

## Discussion

In this study, the Bayesian hierarchical Cox proportional hazards model was adopted to reduce the dimensionality of the omics data as part of the research strategy. Through internal and external validation, the prediction of the prognosis model for lung adenocarcinoma performed well and its performance was better than that of models reported by others^22,23^. The clinical factors and 14-gene signature we identified through the prediction model are basically consistent with previous reports. Interestingly, we also found that CTPS2 and DARS2 which never be reported were associated with the increasing death risk of lung adenocarcinoma.

In this study, 14 prognostic genes were combined with clinical factors, and the final prognosis model for lung adenocarcinoma was constructed. In the training set and validation set, the *C*-index of the model reached 0.733 and 0.735, respectively, which indicates that the performance of the model is reliable. In a previous study of lung adenocarcinoma based on mRNA data from the TCGA database, Hugo Gómez-Rueda et al. constructed a prognostic model through LASSO regression and reported a lower *C*-index (*C*-index = 0.72)^22^. In another study, even with the combination of four omics datasets (mRNA, miRNA, DNA methylation and copy number variations) analysed by deep learning, the performance of the model was not as good as ours (*C*-index = 0.65)^23^. Although a study on early-stage lung adenocarcinoma further improved the *C*-index from 0.728 to 0.756 by adding BRCA1 and ERBB3 into the model, this method has not been verified internally and externally^24^.

Our model was developed with a combination of LASSO Cox and Bayesian methods, which has several advantages over LASSO Cox. This method was also reported to be more accurate than the LASSO Cox regression model for coefficient estimation and prognosis prediction^25^. Additionally, the spike-and-slab prior used in the fitting of the Bayesian hierarchical Cox proportional hazards model can produce different shrinkages for different predictors, reduce the noise from irrelevant predictors and improve the accuracy of coefficient estimation and prediction^12^. The EM cyclic coordinate descent algorithm can make the convergence speed of the model faster on the premise of identifying important factors, which is an important element affecting the generalization of the model^26^.

Furthermore, using the novel Bayesian hierarchical Cox proportional hazards model, most of the 14 prognostic genes we found can be explained in terms of basic study and population study. It is reported that CPS1, IGFBP3, MCM5, MCM7, NT5E, PLK1, PTTG1, SERPINB5, TXNRD1 and TYMS were associated with the prognosis of lung adenocarcinoma or non-small cell lung cancer, which was also included in our 14 genetic findings^27–36^. Basic studies found that NME4 and POLR3G were related to tumorigenesis and the progression of lung adenocarcinoma^37,38^. Our study also revealed that high expression of NME4 and POLR3G may adversely affect the poor prognosis of lung adenocarcinoma. To the best of our knowledge, there were no biological mechanism studies about CTPS2 and DARS2 that affect the tumorigenesis and progression of lung adenocarcinoma. The relationship between CTPS2 and DARS2 and the prognosis of lung adenocarcinoma has not been studied.

The protein encoded by CTPS2 is an important enzyme belonging to the CTP synthase family, which regulates cytosine nucleotide synthesis and provides the necessary precursors for RNA and DNA synthesis^39^. As early as 1978, researchers discovered that cancer cells with increased cell proliferation capabilities also showed increased CTP synthase activity, especially hepatocellular carcinoma cells^40^. Another study also reported that CTPS2 is a key gene that affects the prognosis of osteosarcoma^39^. Here, our study also showed that CTPS2 is an important gene for the prognosis of lung adenocarcinoma, it is highly expressed in patients with lung adenocarcinoma, and the prognosis is poor. Based on the above evidence, it is reasonable to suggest that the CTPS2 gene may be a new potential target for selective chemotherapy of lung adenocarcinoma. However, the mechanism of CTPS2 in lung adenocarcinoma is not clear, and more research is necessary.

The protein encoded by DARS2 is a critical mitochondrial enzyme belonging to the class-II aminoacyl-tRNA synthetase family, which is important for the mitochondrial unfolded protein response^41^. The relationship between the DARS2 gene and leukoencephalopathy with brain stem and spinal cord involvement and lactate elevation has been studied most frequently^42^. The first report on the relationship between DARS2 and cancer was in 2017, in which it was reported that DARS2 can promote the development of hepatocellular carcinoma by accelerating the cell cycle and reducing apoptosis^43^. Our study also showed that with an increased expression of DARS2, the death risk of patients with lung adenocarcinoma gradually elevated. We infer that DARS2 also affects the prognosis of lung adenocarcinoma by accelerating cell cycle progression and attenuating cell apoptosis, but further research is necessary to verify its function.

In summary, we constructed a prognosis prediction model of lung adenocarcinoma that the performance of the model is well and drew a nomogram, which provided a powerful tool for clinicians to predict the prognosis of lung adenocarcinoma patients. What's more, the main innovation of our study is the application of the Bayesian hierarchical Cox proportional hazards model for the reduction of omics data dimensionality to screen for prognostic genes. However, there are some limitations to the study. First, though our study adopted a new strategy of combining omics data with clinical characteristics, there are many possible research strategies in this field. It is a major challenge to determine which procedure is the best for model construction. To solve these problems, we should conduct some simulations and case studies in the future to explore the best research strategy for cancer prognosis prediction. In addition, there may be interactions and more complex nonlinear relationships between genes, which were unfortunately not analyzed in this study. Therefore, whether this method can be used to identify complex nonlinear relationships will be a focus of future research. Finally, although the statistical analysis was used to test the expression of genes that have not been fully explored in lung adenocarcinoma, we also expect to verify the expression of related genes by in vitro and in vivo experiments and explain the important role of CTPS2 and DARS2 in lung adenocarcinoma in further study.

## Conclusions

The Bayesian hierarchical Cox proportional hazards model is a highly effective and alternative method for dealing with high-dimensional omics data when constructing cancer prediction and prognosis models. CTPS2 and DARS2 are new signatures affecting the prognosis of lung adenocarcinoma and may be potential new treatment targets.

## Supplementary Information

Supplementary Information.
