# Supplementary material for: A novel 14-gene signature for overall survival in lung adenocarcinoma based on the Bayesian hierarchical Cox proportional hazards model
Source: Sci Rep. 2022 Jan 7;12:27. doi: 10.1038/s41598-021-03645-6 (PMC8741994; doi:10.1038/s41598-021-03645-6)
Supplement: Supplementary file 1 — Supplementary Information. [file 41598_2021_3645_MOESM1_ESM.docx]

**Supplementary**

Table S1 Gene sets enrichment in phenotype: tumour

| Gene set name | Site | Leading edge subset | NES | NOM *p*-val | FDR *q*-val |
| --- | --- | --- | --- | --- | --- |
| One carbon pool by folate | 17 | 8 | 1.726 | 0.006 | 0.095 |
| Cell cycle | 124 | 42 | 1.700 | 0.034 | 0.099 |
| Base excision repair | 33 | 20 | 1.880 | 0.002 | 0.102 |
| Aminoacyl-tRNA biosynthesis | 22 | 12 | 1.727 | 0.041 | 0.118 |
| DNA replication | 36 | 25 | 1.640 | 0.023 | 0.124 |
| Homologous recombination | 28 | 18 | 1.595 | 0.042 | 0.133 |
| Mismatch repair | 23 | 13 | 1.734 | 0.014 | 0.149 |
| Alanine, aspartate and glutamate metabolism | 30 | 10 | 1.601 | 0.010 | 0.155 |
| Pyrimidine metabolism | 98 | 45 | 1.743 | 0.011 | 0.205 |
| p53 signalling pathway | 67 | 16 | 1.474 | 0.049 | 0.209 |


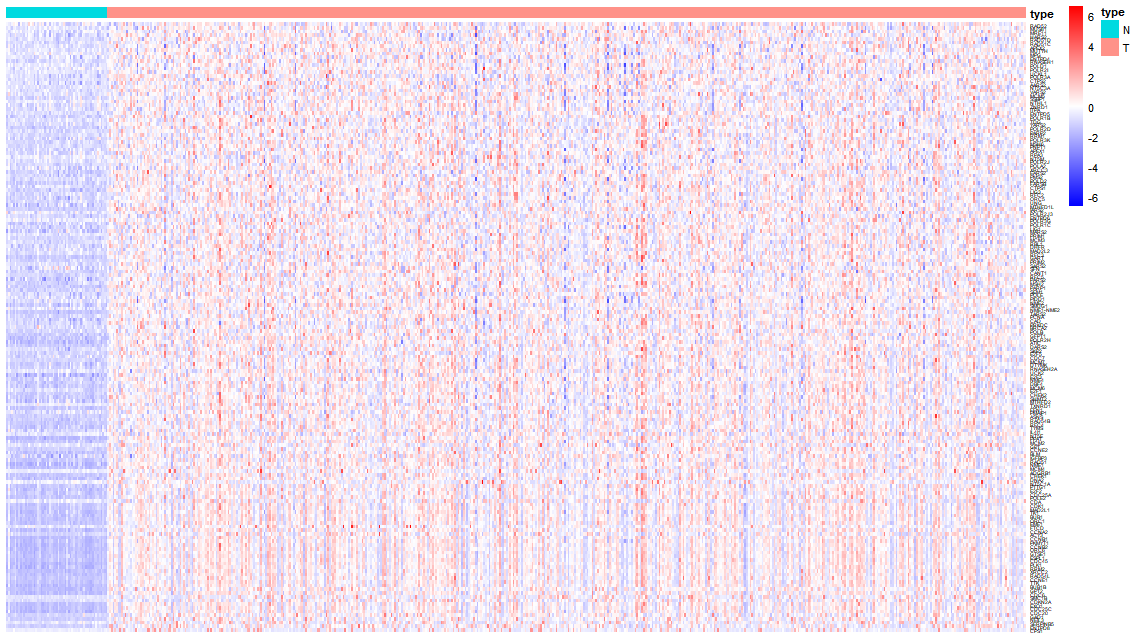
Figure S1 Heatmap of 165 mRNAs in the tumour and normal groups.


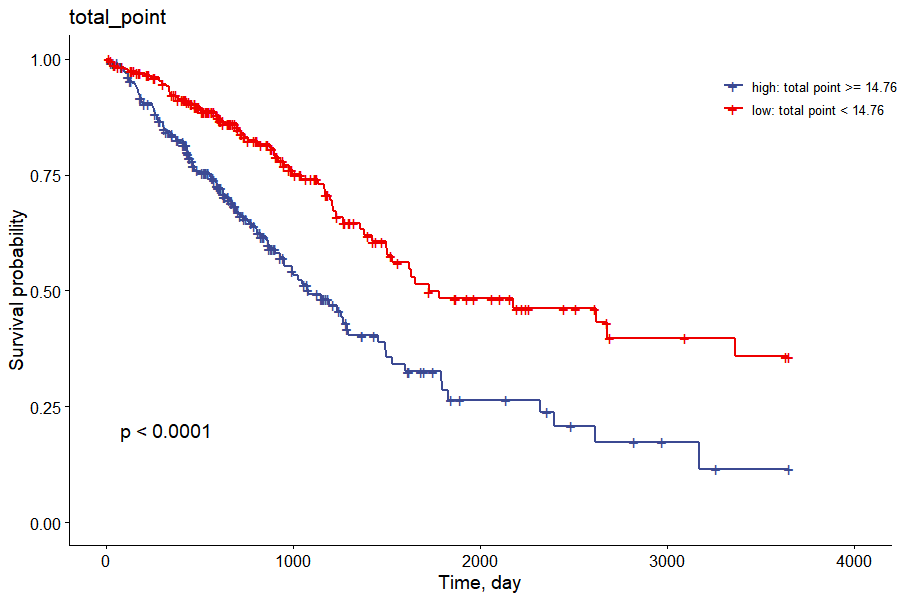


Figure S2 Kaplan-Meier curve of TCGA-LUAD survival data for high and low groups (the median total point=14.76) with *P*<0.001.
